# An MRI-based radiomics nomogram for detecting cervical esophagus invasion in hypopharyngeal squamous cell carcinoma

**DOI:** 10.1186/s40644-023-00642-y

**Published:** 2023-12-15

**Authors:** Meng Qi, Yan Sha, Duo Zhang, Jiliang Ren

**Affiliations:** 1 Department of Radiology, Eye & ENT Hospital, Fudan University, No.83 Fenyang Road, Shanghai, 200030 China; 2 Department of Otolaryngology–HNS, Eye & ENT Hospital, Fudan University, No.83 Fenyang Road, Shanghai, 200030 China; 3 Department of Radiology, Shanghai Ninth People’s Hospital, Shanghai Jiao Tong University School of Medicine, No.639 Zhizaoju Road, Shanghai, 200010 China

**Keywords:** Magnetic resonance imaging, Radiomics, Hypopharyngeal squamous cell carcinoma, Cervical esophagus invasion

## Abstract

**Background:**

Accurate detection of cervical esophagus invasion (CEI) in HPSCC is challenging but crucial. We aimed to investigate the value of magnetic resonance imaging (MRI)-based radiomics for detecting CEI in patients with HPSCC.

**Methods:**

This retrospective study included 151 HPSCC patients with or without CEI, which were randomly assigned into a training (*n* = 101) or validation (*n* = 50) cohort. A total of 750 radiomics features were extracted from T2-weighted imaging (T2WI) and contrast-enhanced T1-weighted imaging (ceT1WI), respectively. A radiomics signature was constructed using the least absolute shrinkage and selection operator method. Multivariable logistic regression analyses were adopted to establish a clinical model and a radiomics nomogram. Two experienced radiologists evaluated the CEI status based on morphological findings. Areas under the curve (AUCs) of the models and readers were compared using the DeLong method. The performance of the nomogram was also assessed by its calibration and clinical usefulness.

**Results:**

The radiomics signature, consisting of five T2WI and six ceT1WI radiomics features, was significantly associated with CEI in both cohorts (all *p* < 0.001). The radiomics nomogram combining the radiomics signature and clinical T stage achieved significantly higher predictive value than the clinical model and pooled readers in the training (AUC 0.923 vs. 0.723 and 0.621, all *p* < 0.001) and validation (AUC 0.888 vs. 0.754 and 0.647, all *p* < 0.05) cohorts. The radiomics nomogram showed favorable calibration in both cohorts and provided better net benefit than the clinical model.

**Conclusions:**

The MRI-based radiomics nomogram is a promising method for detecting CEI in HPSCC.

## Introduction

Head and neck cancers are the seventh most common type of malignancy worldwide [1]. Hypopharyngeal squamous cell carcinoma (HPSCC) constitutes 3–5% of all head and neck cancers [1, 2]. Primary HPSCCs are often locally advanced at the time of diagnosis and frequently combined with the invasion of nearby anatomical structures [3]. Among these structures, cervical esophagus invasion (CEI) is an important factor distinguishing between T2 and T3/4 stages according to the 8th edition of American Joint Committee on Cancer (AJCC) staging system [4]. In addition, it has been established that the existence of CEI is strongly correlated with poor prognosis of the patients with HPSCC [5]. Thus, accurate determination of CEI is crucial in making clinical decisions for HPSCCs. At present, esophagoscopy is listed as one of the recommended procedures for detecting CEI in newly diagnosed HPSCC. However, it has numerous contraindications in clinical applications and may overlook pertinent information regarding submucosal extension [3, 6–8]. Therefore, there is a pressing need to explore alternative approaches that can reliably identify CEI in HPSCCs before treatment.

Magnetic resonance imaging (MRI) is instrumental in the preoperative staging of HPSCC and has been increasingly used to evaluate tumor invasion into surrounding structures [9–11]. However, information derived from MRI commonly refers to some simple traits, such as primary tumor location, and thickness and contrast enhancement of the cervical esophageal wall [3]. In addition, visual evaluation of these conventional morphological features is limited by subjectivity and lack of a consensus. Radiomics, which involves extracting high-throughput quantitative features from medical imaging, can noninvasively provide information about tumor heterogeneity [12–14]. MRI-based radiomics has demonstrated great potential to predict therapeutic response [15], lymph node metastasis [16], and survival [17, 18] for HPSCC. However, to our knowledge, there are no reports documenting whether radiomics could facilitate the detection of CEI in HPSCC.

In this study, we aimed to develop and validate an MRI-based radiomics nomogram for preoperative prediction of CEI in patients with HPSCC.

## Methods

### Patients

**Fig. 1:**
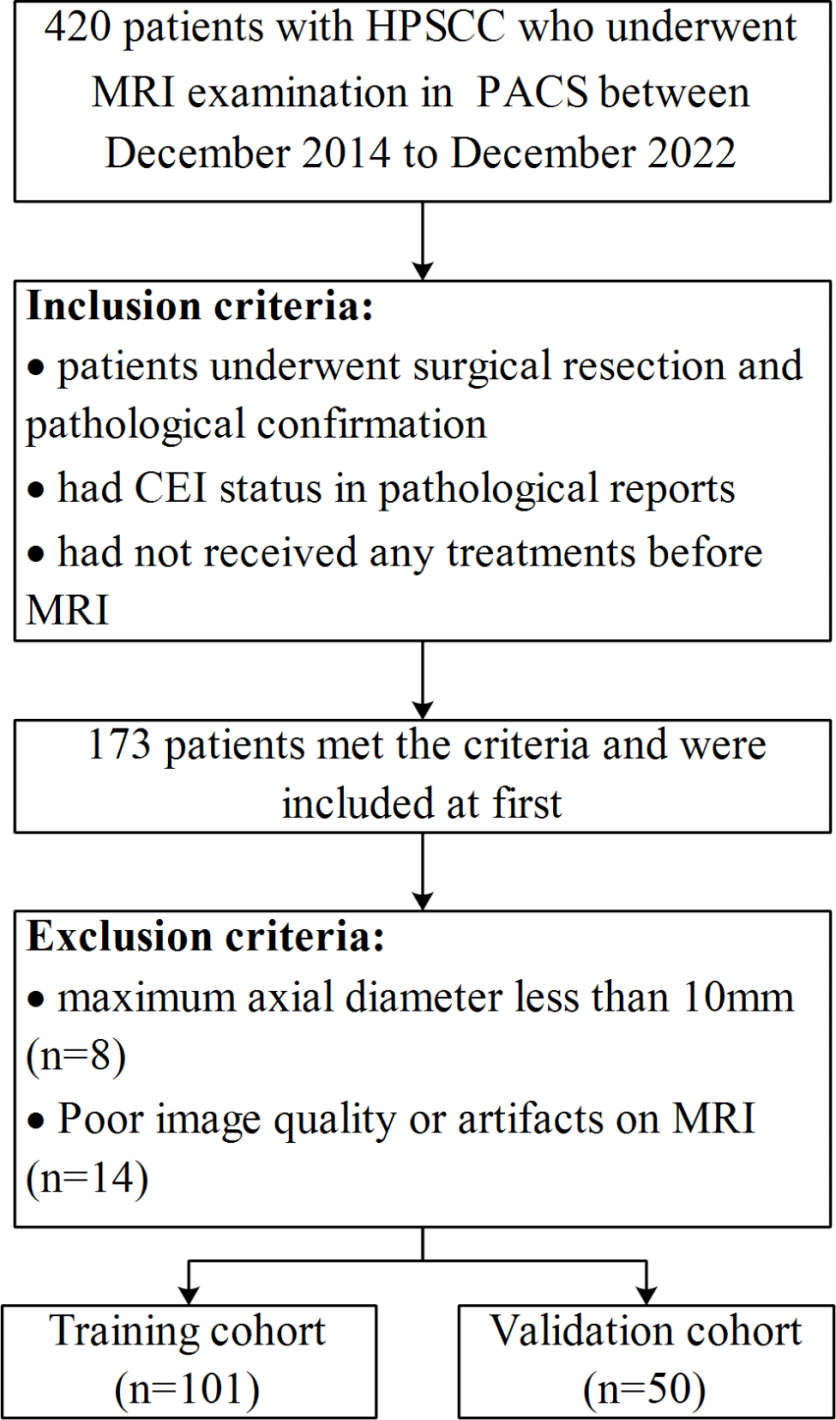
Flowchart of the patient selection process. HPSCC, hypopharyngeal squamous cell carcinoma; PACS, picture archiving and communication system; CEI, cervical esophagus invasion

This retrospective study was approved by the Ethics Review Board of Shanghai Eye & ENT Hospital of Fudan University, and the requirement of informed consent was waived. We thoroughly search the picture archiving and communication system (PACS) of Shanghai Eye & ENT Hospital from December 2014 to December 2022 to retrieve data. Patients were selected or excluded according to the criteria presented in Fig. 1. A total of 151 patients were enrolled in the study and randomly allocated to a training cohort (*n* = 101) or a validation cohort (*n* = 50) at a ratio of 2:1. The clinical characteristics of each patient before treatment including gender, age, primary site, maximum diameter, clinical T stage, clinical N stage, and clinical TNM stage were recorded. The gold standard for assessing CEI is determined by the postoperative pathological findings.

### MRI acquisition

MRI examinations was performed on a 3.0 T scanner (Magnetom Verio, Siemens Medical, Erlangen, Germany) using a 12-channel head and neck array coil. The axial fat-suppressed T2-weighted imaging (T2WI) and contrast-enhanced T1-weighted imaging (ceT1W) were used for analysis. The MRI acquisition parameters were as follows: axial T2WI (repetition time [TR] / echo time [TE], 4000 ms / 99 ms; matrix, 640 × 592; field of view, 220 mm × 220 mm; thickness, 6 mm; gap, 0.6 mm) and axial ceT1W (TR / TE, 384 ms / 9 ms; matrix, 640 × 592; field of view, 220 mm × 220 mm; thickness, 6 mm; gap, 0.6 mm). A standard dose of 0.1 mmol/kg of gadopentetate dimeglumine (Magnevist, Bayer Healthcare Pharmaceuticals, Berlin, Germany) was administered for ceT1WI.

### Tumor segmentation and image processing

**Fig. 2:**
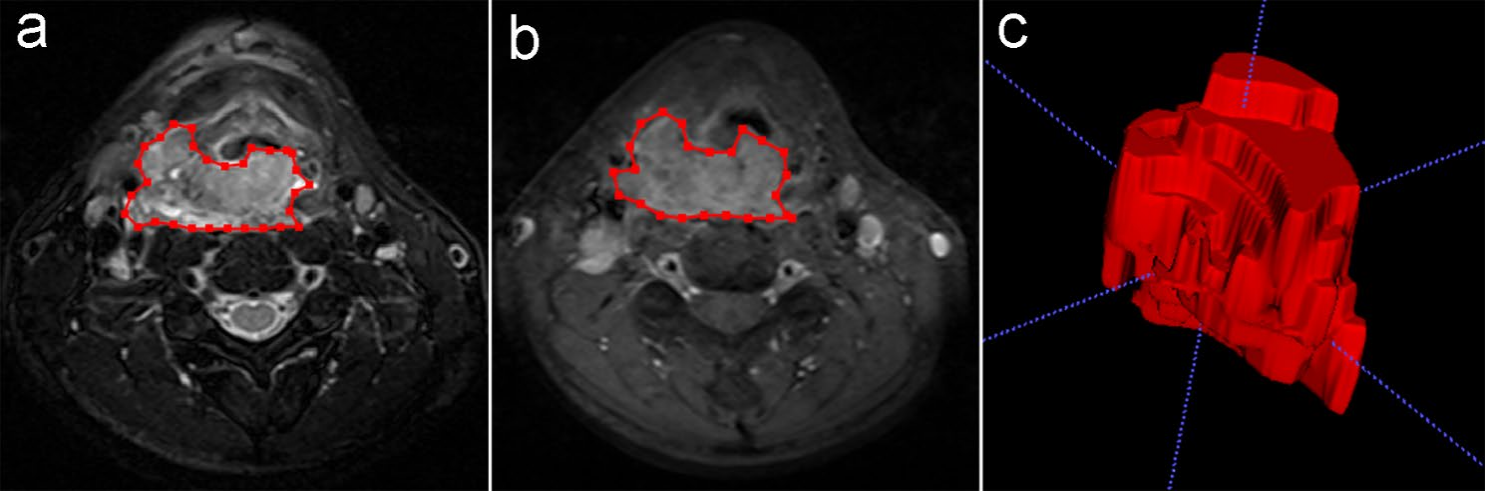
Tumor segmentation of hypopharyngeal squamous cell carcinoma. The segmentation was performed on T2-weighted imaging (**a**) and contrast-enhanced T1-weighted imaging (**b**). By drawing regions of interest slice by slice, a region of interest covering the entire tumor (**c**) was acquired

Tumor segmentation was performed with the open-source ITK-SNAP software (version 3.6.0; www.itk-snap.org) on T2WI and ceT1WI images independently. Three-dimensional regions of interest (ROIs) were manually drawn slice by slice to cover the entire tumor by Radiologist 1 (M.Q.) with 6 years of experience of head-and-neck MRI interpretation. Subsequently, 30 randomly selected lesions were segmented by Radiologist 2 (J.R.) with 8 years of experience of head-and-neck MRI interpretation. Dice similarity coefficient (DSC) was calculated to evaluate the interobserver’s agreement of tumor segmentation between two radiologists. The radiologists were blinded to clinical information and histopathologic results regarding CEI status. For the hypopharynx/esophagus junction, the areas of esophageal thickening with significant enhancement on contrast-enhanced T1WI were categorized as the regions affected by the tumor, and were consequently incorporated for analysis. In addition, contrast-enhanced T1WI was required to be referenced in order to ascertain the tumor boundary on T2WI. Tumor segmentation is illustrated in Fig. 2.

Three image-processing methods were conducted on all images before feature extraction. First, the in-plane resolution was rescaled to 1 × 1 mm^2^; second, the gray-level was normalized using µ ± 3σ technique (scale, 100); and finally, gray-level discretization was completed with the bin count set as 64.

### Radiomic-feature extraction

Radiomics features were extracted from T2WI and ceT1WI images using PyRadiomics, an open-source Python package (version 3.0.1; www.radiomics.io). The radiomics features included 14 shape- and size-based features, 17 first-order histogram features, and 75 textural features. Five classes of textural features were extracted: gray-level co-occurrence matrix (GLCM), gray-level dependence matrix (GLDM), gray-level run-length matrix (GLRLM), gray-level size zone matrix (GLSZM), and neighboring gray-tone difference matrix (NGTDM). The original, Laplacian of Gaussian (LoG)-filtered (values of 1, 3, and 5 mm) and wavelet-transformed (four different combinations of high- and low-frequency bands) images were separately used for calculating the histogram and textural features. In total, 750 radiomics features were obtained from each sequence. Detailed information on the radiomics features have been described elsewhere (pyradiomics.readthedocs.io/en/latest/index.html).

### Feature selection and development of a radiomics signature

To evaluate the interobserver reproducibility, intraclass correlation coefficients (ICCs) were calculated for each radiomics feature. Only the features with satisfactory interobserver reproducibility (ICC ≥ 0.8) were retained. Then, the collinearity among features was evaluated using Spearman’s correlation coefficients (*r*). If a pair of features had high collinearity (*r* > 0.8), the one with higher collinearity with the remaining features was excluded. Subsequently, Mann–Whitney U tests were conducted for the non-redundant features. The features that differed significantly between the two groups with and without CEI were screened out for further analysis. Finally, the most significant features were selected to construct a radiomics signature using the least absolute shrinkage and selection operator (LASSO) method with 10-fold cross-validation. The radiomics score for each patient was calculated by a linear combination of the features weighted by their respective coefficients.

### Development of a clinical model and radiomics nomogram

The significant clinical characteristics alone and with the radiomics signature were entered into multivariate logistic regression analysis to respectively establish the clinical model and radiomics model for the training cohort. A nomogram based on the radiomics model was constructed. The performance of the two models for detecting CEI were tested in the validation cohort.

### MRI visual assessment

Two radiologists (M.Q. and J.R) jointly reviewed all MRI scans to evaluate the status of CEI before tumor segmentation. Any disagreement was resolved through consultation. At approximately six-month intervals, two radiologists performed individual assessments of the MRI scans. The classification outcomes from two readers were documented for both the pooled and individual assessments. The radiologists were blinded to the clinical information and histopathologic details.

### Statistical analyses

Statistical analyses were performed using R software (version 3.5.2; www.r-project.org). The differences in clinical characteristics and radiomics features between the patients with and without CEI were assessed. All variables were compared by the χ^2^ test or Mann–Whitney U test, where appropriate. The kappa value was calculated to assess the diagnosis consistency of CEI between two radiologists, where the kappa value of 0.75 to 1.00 showed almost perfect consistency, 0.4 to 0.75 as moderate consistency, and 0 to 0.40 as a poor or no consistency [19]. Receiver operating characteristic (ROC) curve analysis was used to evaluate the predictive performance of the significant variables, prediction models, and pooled readers. The area under the curve (AUC), accuracy, sensitivity, and specificity were derived. The AUC values of the radiomics nomogram for the two cohorts were compared with those of the clinical model and pooled readers using the DeLong method. The Hosmer–Lemeshow test was used to assess the calibration of the radiomics nomogram. Decision curve analysis was performed to quantify the net benefit from the use of the clinical model and radiomics nomogram at different threshold probabilities. *p* < 0.05 was considered significant.

## Results

### Patient characteristics

The baseline characteristics of all patients are summarized in Table 1. The rates of CEI were 38.6% (39 of 101) and 52% (26 of 50) in the training and validation cohorts, respectively, whereas no difference was found between the two cohorts (*p* = 0.165). Significant differences in primary site, maximum diameter, and clinical T stage were observed between the patients with and without CEI in the training cohort. After multiple logistic regression analysis, primary site (*p* = 0.015) and clinical T stage *(p* = 0.033*)* were confirmed as independent predictors for CEI and were used to construct the clinical model (Table 2). ROC curve analysis showed that the AUCs for primary site and clinical T stage were 0.652 and 0.647, respectively, in the training cohort, and 0.598 and 0.692, respectively, in the validation cohort (Table 3).

**Table 1:** Clinical characteristics and radiomics score of patients

	Training cohort (n = 101)			Validation cohort (n = 50)		
	Without CEI (n = 62)	With CEI (n = 39)	*p value*	Without CEI (n = 24)	With CEI (n = 26)	*p value*
Gender						
Female	4 (6.5%)	2 (5.1%)	1	1 (4.2%)	1 (3.8%)	1
Male	58(93.5%)	37 (94.9%)		23 (95.8%)	25 (96.2%)	
Age (years)	61 (56, 65)	58 (55, 62)	0.056	61 (56, 66)	61 (53, 67)	0.899
Primary site			0.012			0.201
Pyriform sinus	38 (61.3%)	12 (30.8%)		13 (54.2%)	8 (30.8%)	
Posterior pharyngeal wall	15 (24.2%)	17 (43.6%)		6 (25.0%)	12 (46.2%)	
Postcricoid region	9 (14.5%)	10 (25.6%)		5 (20.8%)	6 (23.1%)	
Maximum diameter (mm)	33 (28, 39)	38 (23, 44)	0.001	30 (24, 36)	41 (36, 48)	< 0.001
Clinical T stage			0.001			0.005
T1-2	23 (37.1%)	3 (7.7%)		12 (50.0%)	3 (11.5%)	
T3-4	39 (62.9%)	36 (92.3%)		12 (50.0%)	23 (88.5%)	
Clinical N stage			0.830			0.164
N0	16 (25.8%)	10 (25.6%)		7 (29.2%)	3 (11.5%)	
N1-3	46 (74.2%)	29 (74.4%)		17 (70.8%)	23 (88.5%)	
Clinical TNM stage			0.122			0.467
I-II	10 (16.1%)	2 (5.1%)		3 (12.5%)	6 (23.1%)	
III-IV	52 (83.9%)	37 (94.9%)		21 (87.5%)	20 (76.9%)	
Radiomics score	-1.04 (-1.61, -0.41)	0.24 (-0.16, 0.57)	< 0.001	-1.30 (-1.65, -0.30)	0.45 (-0.10, 0.89)	< 0.001

CEI, cervical esophagus invasion

Data are expressed as median (interquartile range) or number (percentage)

**Table 2:** Risk factors for cervical esophagus invasion in hypopharyngeal squamous cell carcinoma

Variable	Radiomics Model		Clinical Model	
	Odds ratio	*p* value	Odds ratio	*p* value
Primary sites	0.88 (0.34, 2.23)	0.781	2.09 (1.16, 3.78)	0.015
Clinical T stage	10.02 (1.55, 65.04)	0.016	4.64 (1.13, 19.11)	0.033
Maximum diameter	0.93 (0.89, 1.02)	0.108	1.05 (0.99, 1.10)	0.084
Radiomics signature	61.24 (9.54, 393.08)	< 0.001	NA	NA

Data are results of the multivariable regression analysis. Data in parentheses are 95% confidence intervals

NA, not available

**Table 3:** Diagnostic performance of the significant predictors in the training and validation cohorts

	AUC	Accuracy	Sensitivity	Specitivity
Training cohort				
Primary site	0.652 (0.550, 0.754)	64.4 (63.9, 64.8)	69.2 (54.7, 83.7)	61.3 (49.2, 73.4)
Clinical T stage	0.647 (0.573, 0.721)	58.4 (57.9, 58.9)	92.3 (83.9, 100)	37.1 (25.1, 49.1)
Radiomics signature	0.916 (0.866, 0.967)	80.2 (79.9,80.5)	97.4 (92.5, 100)	69.4 (57.9, 80.8)
Validation cohort				
Primary sites	0.598 (0.446, 0.749)	62.0 (61.1, 62.9)	69.2 (51.5, 87.0)	54.2 (34.2, 74.1)
Clinical T stage	0.692 (0.572, 0.812)	70.0 (69.2,70.8)	88.5 (76.2, 100)	50.0 (30.0, 70.0)
Radiomics signature	0.865 (0.762, 0.969)	82.0 (81.4, 82.6)	84.6 (70.7, 98.5)	79.2 (62.9, 95.4)

Data are presented as percentages, except AUC; 95% confidence intervals are included in parentheses

AUC, area under the curve

### Development of a radiomics signature

**Fig. 3:**
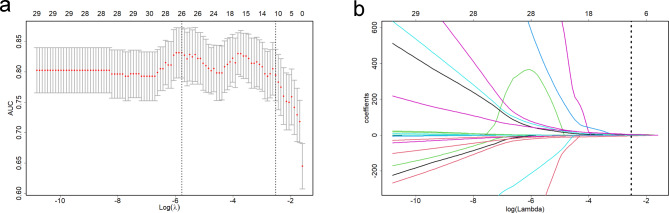
Radiomic feature selection by least absolute shrinkage and selection operator (LASSO) logistic regression. (**a**) Selection of tuning parameter (λ) in the LASSO model using 10-fold cross-validation. Dotted vertical lines were drawn at the optimal values by using the minimum criteria and 1 standard error of the minimum criteria (the 1-standard error criteria). The optimal log (λ) of -2.53 was chosen. (**b**) LASSO coefficient profiles of the 31 features. A vertical line was plotted at the optimal log (λ), which resulted in 11 features with non-zero coefficients

The average DSC values obtained by the two radiologists were 81.8% ± 7.06% and 82.2% ± 5.27% for the delineations generated on T2WI and ceT1WI, respectively. In total, 65.7% (493/750) of T2WI features and 74.3% (557/750) of ceT1WI features showed satisfactory interobserver agreement (ICC ≥ 0.8). After the collinearity analysis, 52 T2WI and 54 ceT1WI features were retained. In the training cohort, significant differences were observed in 13 T2WI and 18 ceT1WI features between the two groups with and without CEI. Finally, the LASSO regression identified 11 features (5 T2WI and 6 ceT1WI features) with non-zero coefficients that were used to develop a radiomics signature (Table 4; Fig. 3). The radiomics score showed a significant difference between the two groups in both cohorts (all *p* < 0.001, Table 1). The AUC values for the radiomics signature were 0.916 and 0.865 in the training and validation cohorts, respectively (Table 3).

**Table 4:** LASSO coefficients of the selected features for the radiomics signature

Sequence	Image type	Feature class	Feature name	LASSO coefficient
T2WI	Original	Shape	MajorAxisLength	1.89E-03
T2WI	Original	GLSZM	LargeAreaHighGrayLevelEmphasis	9.80E-05
T2WI	LoG_3mm_	GLCM	Imc1	2.06E + 00
T2WI	LoG_3mm_	GLRLM	RunEntropy	-6.36E-01
T2WI	Wavelet_HL_	GLDM	DependenceEntropy	-7.39E-01
ceT1WI	Original	Shape	Flatness	-4.20E + 00
ceT1WI	LoG_3mm_	Histogram	90Percentile	1.27E-03
ceT1WI	LoG_3mm_	GLCM	Imc1	2.51E + 00
ceT1WI	LoG_5mm_	NGTDM	Busyness	4.72E + 00
ceT1WI	Wavelet_HH_	GLDM	LargeDependenceHighGrayLevelEmphasis	4.75E-04
ceT1WI	Wavelet_LL_	GLCM	Imc1	4.05E + 00
			(Intercept)	9.50E + 00

GLCM, gray-level co-occurrence matrix; GLRLM, gray-level run-length matrix; GLDM, gray-level dependence matrix; GLSZM, gray-level size zone matrix; NGTDM, neighboring gray-tone difference matrix; LASSO, least absolute shrinkage and selection operator

### Development of a radiomics nomogram

**Fig. 4:**
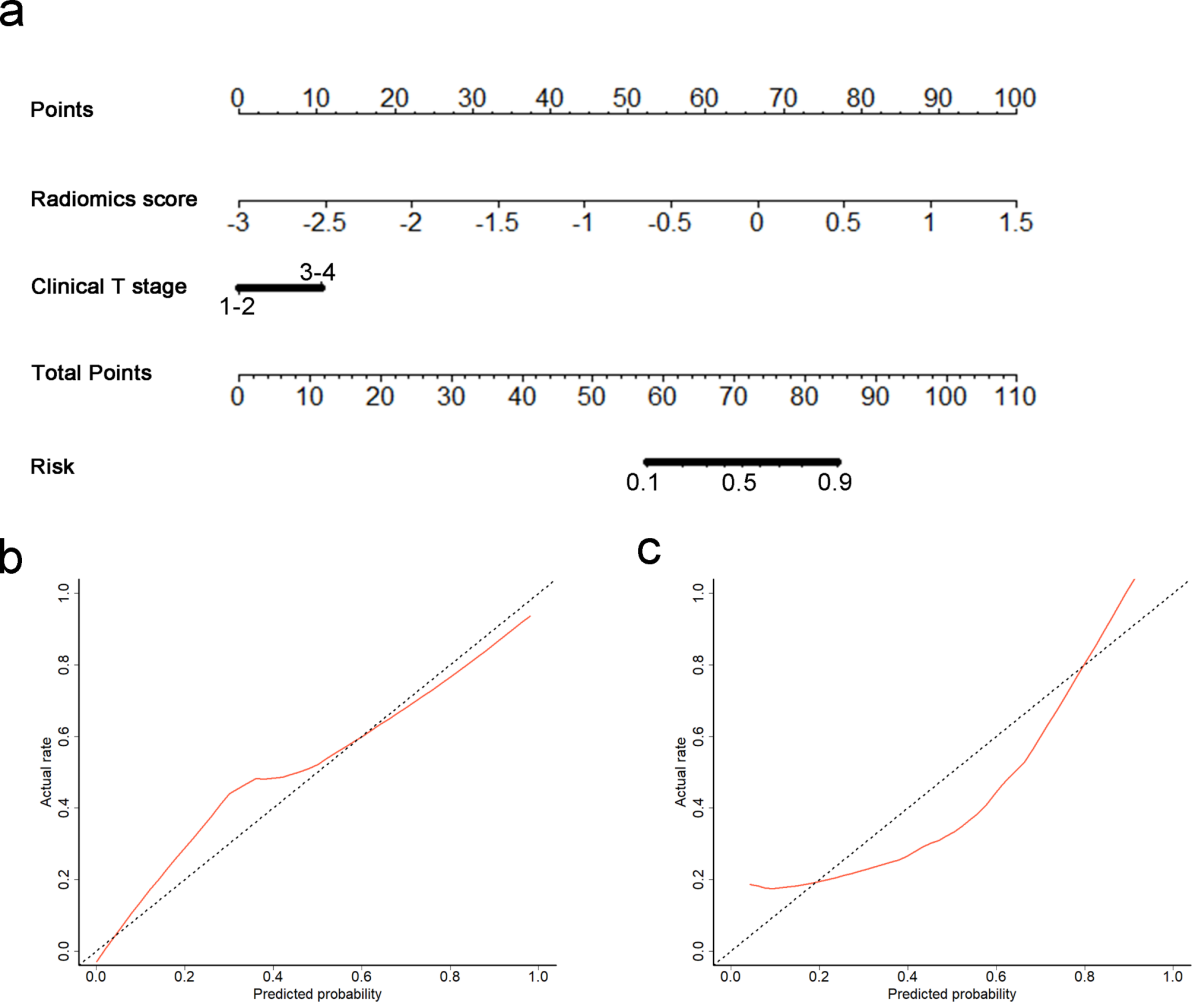
Radiomics nomogram developed with calibration curves. (**a**) A radiomics nomogram was developed in the training cohort, with radiomics signature and clinical T stage incorporated. Calibration curves of the radiomics nomogram in the training (**b**) and validation (**c**) cohorts

When the radiomics signature and clinical characteristics were incorporated, the radiomics signature (*p* < 0.001) and clinical T stage (*p* = 0.016) were identified as independent predictors and were used to construct a radiomics nomogram (Table 2; Fig. 4a). The radiomics nomogram exhibited good calibration and yielded non-significant results in the training and validation cohorts (*p* = 0.526 and 0.969) (Fig. 4b and c).

### Performance of the prediction models and readers

**Fig. 5:**
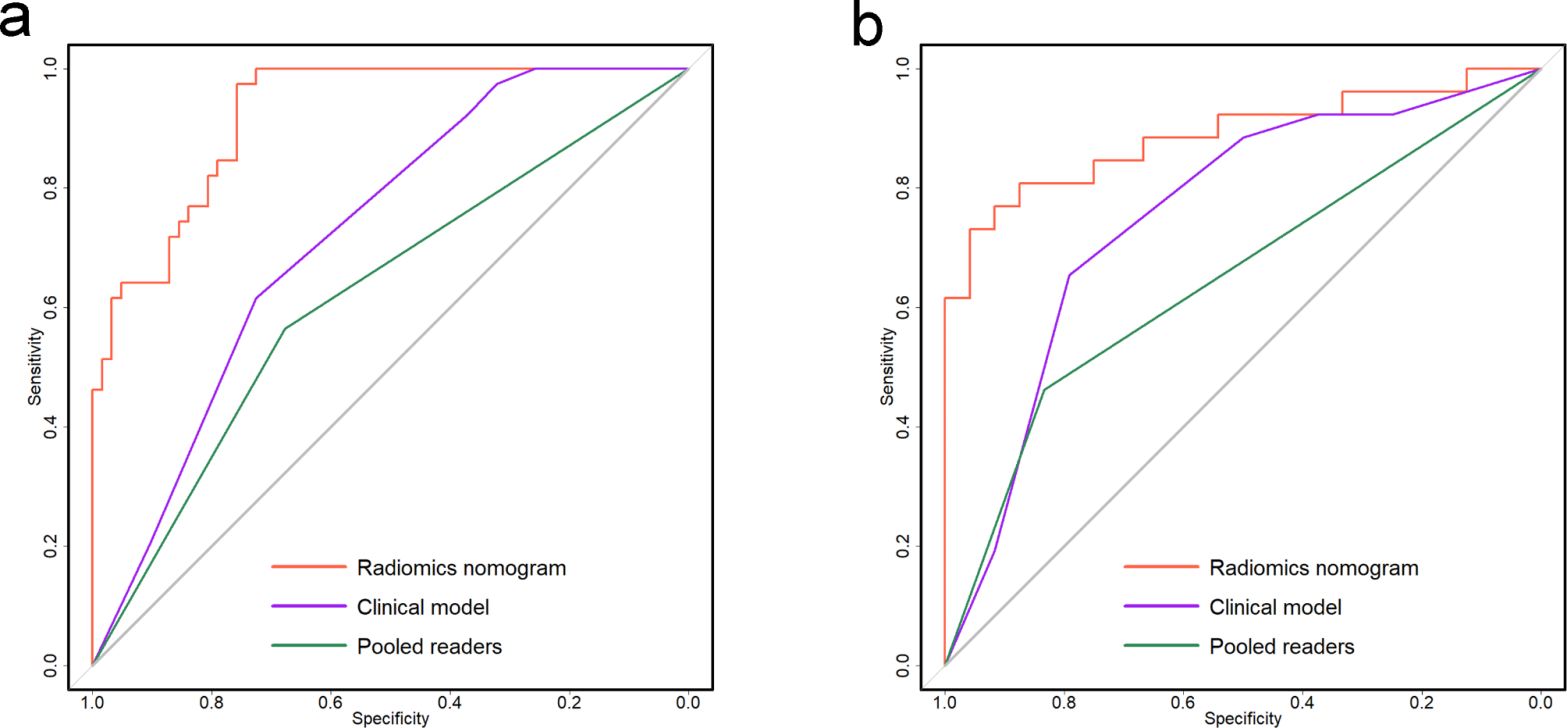
Receiver operating characteristic curves of the radiomics nomogram, clinical model, and pooled readers in the training (**a**) and validation (**b**) cohorts

**Fig. 6:**
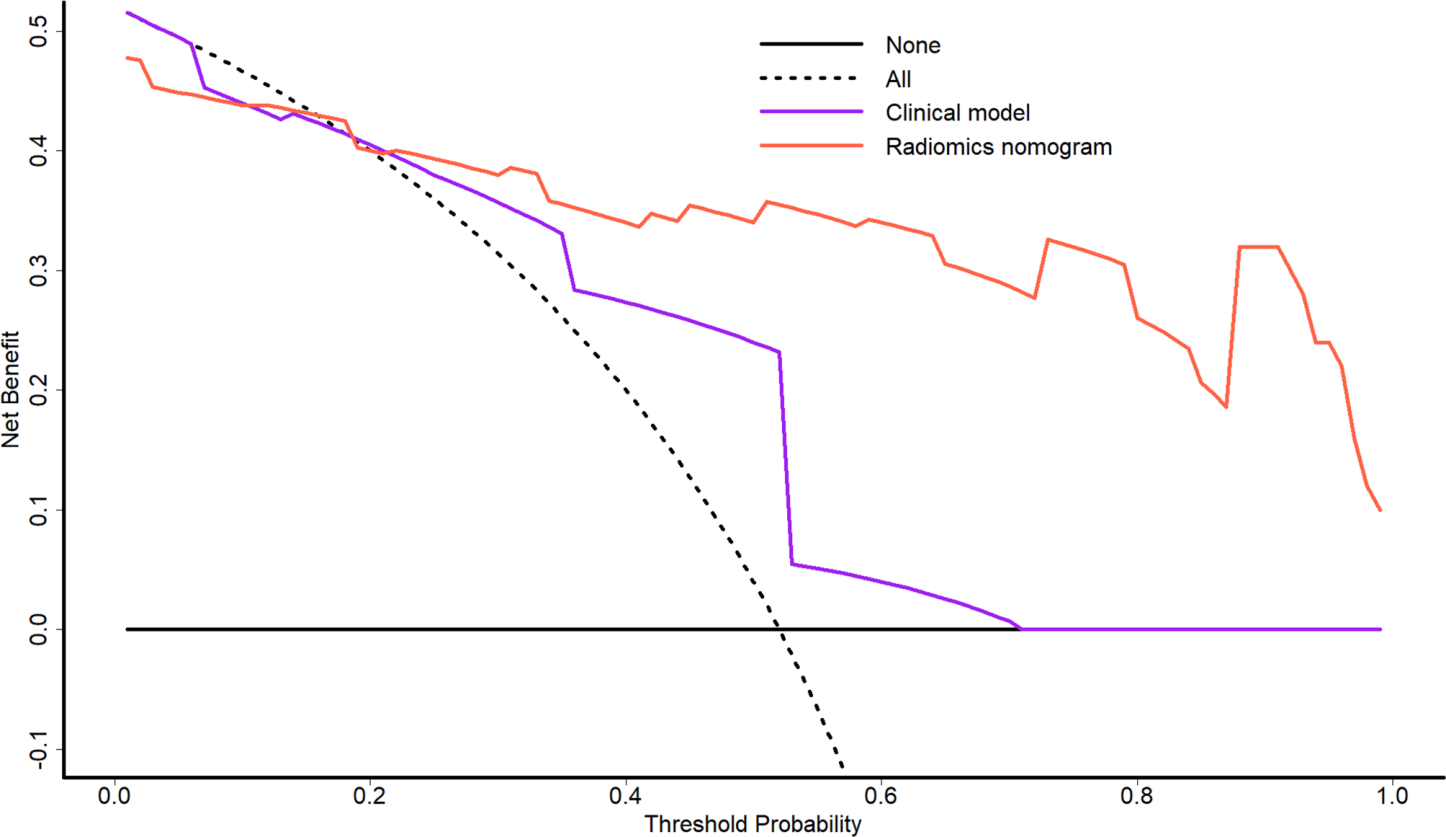
Decision curve analysis for the clinical model and radiomics nomogram. The y-axis indicates the net benefit and the x-axis indicates threshold probability. The radiomics nomogram had a higher overall net benefit in detecting cervical esophagus invasion (CEI) compared with the clinical model and simple diagnoses such as all patients with or without CEI across the majority of the range of reasonable threshold probabilities in the validation cohort

During the individual assessment of CEI status, Radiologist 1 and Radiologist 2 attained accuracies of 62.4% and 63.4% respectively in the training cohort, while achieving accuracies of 62.0% and 66.0% respectively in the validation cohort. When evaluating the diagnostic consensus for CEI between the two radiologists, there was moderate agreement with a kappa value of 0.571. In the training cohort, the clinical model and the pooled readers achieved AUCs of 0.723 and 0.621, respectively and accuracies of 68.3% and 63.4%, respectively. In the validation cohort, the clinical model and the pooled readers achieved AUCs of 0.754 and 0.647, respectively and accuracies of 72.0% and 64.0%, respectively. In the training and validation cohorts, the radiomics nomogram showed the highest discrimination between the patients with and without CEI, with AUCs of 0.923 and 0.888, respectively, and accuracies of 84.2% and 84.0%, respectively. The Delong test indicated that the performance of the radiomics nomogram was significantly superior to that of the clinical model and pooled readers in both cohorts (*p* < 0.001 and *p* < 0.001, respectively, in the training cohort; *p* = 0.036 and *p* < 0.001 in the validation cohort). The AUC, accuracy, sensitivity, and specificity for each model or pooled readers are listed in Table 5 and the ROC curves are provided in Fig. 5. Finally, decision curve analysis indicated that the radiomics nomogram achieved a higher overall net benefit compared with the clinical model across the majority of the range of reasonable threshold probabilities in the validation cohort (Fig. 6).

**Table 5:** Diagnostic performance of the clinical model, radiomics nomogram, and readers in the training and validation cohorts

	AUC	Accuracy	Sensitivity	Specitivity
Training cohort				
Radiomics nomogram	0.923 (0.876, 0.971)	84.2 (83.9, 84.4)	97.4 (92.5, 100)	75.8 (65.1, 86.5)
Clinical model	0.723 (0.630, 0.817)	68.3 (67.9, 68.7)	61.5 (46.3, 76.8)	72.6 (61.5, 83.7)
Pooled readers	0.621 (0.523, 0.719)	63.4 (62.9, 63.8)	56.4 (40.8, 72.0)	67.7 (56.1, 79.4)
Radiologist 1	0.617 (0.519, 0.716)	62.4 (61.9, 62.8)	59.0 (43.5, 74.4)	64.5 (52.6, 76.4)
Radiologist 2	0.664 (0.574, 0.753)	63.4 (62.9, 63.8)	79.5 (66.8, 92.2)	53.2 (40.8, 65.6)
Validation cohort				
Radiomics nomogram	0.888 (0.792, 0.983)	84.0 (83.5, 84.5)	73.1 (56.0, 90.1)	95.8 (87.8, 100)
Clinical model	0.754 (0.617, 0.891)	72.0 (71.2, 72.8)	65.4 (47.1, 83.7)	79.2 (62.9, 95.4)
Pooled readers	0.647 (0.524, 0.771)	64.0 (63.1, 64.9)	46.2 (27.0, 65.3)	83.3 (68.4, 98.2)
Radiologist 1	0.623 (0.489, 0.758)	62.0 (61.1, 62.9)	53.8 (34.7, 73.0)	70.8 (52.6, 89.0)
Radiologist 2	0.659 (0.525, 0.793)	66.0 (65.1, 66.9)	69.2 (51.5, 87.0)	62.5 (43.1, 81.9)

Data are presented as percentages, except AUC; 95% confidence intervals are included in parentheses

AUC, area under the curve

## Discussion

CEI status is a key factor affecting the making of a treatment plan and prognostic evaluation for patients with HPSCC. In this study, we developed and validated a nomogram incorporating an MRI radiomics signature and clinical T stage for detecting CEI in HPSCC. The proposed radiomics nomogram demonstrated superior performance compared with the clinical model and pooled readers in the training and validation cohorts. In addition, the radiomics nomogram provided more net benefit than the clinical model.

Among the clinical characteristics, maximum diameter was eliminated from predictive models, although it showed a significant difference between HPSCCs with and without CEI. However, clinical T stage was confirmed to be an independent predictor of CEI in clinical and radiomics models. These results may be attributed to the fact that clinical T stage comprehensively contains the information of tumor size, structure invasion, and hemilaryngeal fixation. Consistent with a previous study [5], primary site in the posterior pharyngeal wall was significantly associated with the presence of CEI in the training cohort. In addition, the small sample size may contribute to the non-significant difference of primary site between the two groups in the validation cohort. By comparing the predictive performance of the significant variables, we found that the radiomics signature achieved the best performance with AUCs of 0.916 and 0.865 in the training and validation cohorts, respectively. These results preliminarily confirmed that, compared with the clinical characteristics, the radiomics approach could generate more relevant information related to CEI.

Radiomics was raised based on the hypothesis that the spatial distribution of voxel intensities could precisely reflect the intratumor heterogeneity [20, 21]. It can transform medical images into multi-dimensional quantitative data [22]. In our study, five T2WI and six ceT1WI features were identified by LASSO regression to develop the radiomics signature. Radiomics features from T2WI and ceT1WI can reflect the heterogeneity of tumor water content and blood supply, respectively [17, 23]. Therefore, radiomics analysis based on different imaging sequences could provide supplemental information regarding the prediction of CEI in HPSCC. Among the optimal radiomics features, there was one first-order histogram feature and eight textural features (Table 4). Histogram features mainly describe the appearance frequency of each gray level within the whole ROI [24], whereas textural features can comprehensively describe the spatial distribution of pixel intensity [25–27]. Therefore, these features could capture the global, local, and regional heterogeneity of lesions at different scales [24]. In addition, three, five, and three optimal features were extracted from the original, LoG-filtered, and wavelet-transformed images, respectively (Table 4). The grayscale distribution and variation on different types of images may comprehensively reflect tumor microscopic characteristics, which facilitate monitoring of the tumor biological behavior.

To thoroughly investigate the advantages of MRI radiomics, the radiomics nomogram was compared with a clinical model and pooled assessment by two experienced radiologists. We found that a considerable proportion of patients were misclassified according to visual assessment. Morphological MRI assessment predominantly relies on the experience and capabilities of the radiologists [28]. Moreover, the thickness and enhancement pattern of normal cervical esophageal wall varies among different patients, which can also affect the diagnostic accuracy of radiologists [29]. In addition, it further underscores the significance of developing an objective and quantitative imaging marker for CEI, given the unsatisfactory level of diagnostic concordance (kappa value of 0.571) on CEI status between the two radiologists. Our results showed that the radiomics nomogram (AUC 0.888; Accuracy 84.0%) achieved superior performance than the clinical model (AUC 0.754; Accuracy 72.0%) and pooled readers (AUC 0.647; Accuracy 64.0%) in the validation cohort. In addition, the radiomics nomogram exhibited good calibration in the training and validation cohorts. The decision curve analysis showed that the radiomics nomogram had better clinical utility than the clinical model in the validation cohort. Collectively, these results suggest that the nomogram is a reliable and reproducible tool for detecting CEI in HPSCC. Therefore, our findings could potentially benefit clinical practice for HPSCC in the future.

This study does have several limitations. First, sample selection bias could not be eliminated in this the single-center retrospective study due to the strict enrollment criteria utilized. The value of MRI radiomics in predicting CEI in HPSCC needs to be further confirmed in multi-center and large-scale studies. Second, only whole tumor ROIs were used in this study. As the most critical part for evaluating CEI in HPSCC, the hypopharynx/esophagus junction is deserved to be independently to be analyzed. Third, T1WI radiomic features were not explored due to the challenge in tumor segmentation, some important T1WI information regarding CEI may be leaved out. Fourth, all the ROIs were manually delineated, which is time-consuming and has interobserver variation. Therefore, an automatic segmentation approach should be explored in the future. Finally, the methods used in this study need improvement; additional machine learning or deep learning techniques also warrant investigation.

## Conclusion

In conclusion, our MRI-based radiomics nomogram showed good diagnostic efficiency in detecting the CEI status of HPSCC. The nomogram may provide added value for clinical decision-making and prognostic evaluation for patients with HPSCC.

## Data Availability

The datasets used and/or analyzed during the current study are available from the corresponding author on reasonable request.

## Abbreviations

AUC: Area under the curve
CEI: Cervical esophagus invasion
ceT1WI: Contrast-enhanced T1-weighted imaging
DSC: Dice similarity coefficient
HPSCC: Hypopharyngeal squamous cell carcinoma
ICC: Intraclass correlation coefficient
LASSO: Least absolute shrinkage and selection operator
LoG: Laplacian of Gaussian
MRI: Magnetic resonance imaging
PACS: Picture archiving and communication system
ROC: Receiver operating characteristic
ROI: Region of interest
T2WI: T2-weighted imaging
TE: Echo time
TR: Repetition time
